# Unraveling the mechanism of flower color variation in *Brassica napus* by integrated metabolome and transcriptome analyses

**DOI:** 10.3389/fpls.2024.1419508

**Published:** 2024-06-12

**Authors:** Cheng Cui, Ka Zhang, Liang Chai, Benchuan Zheng, Jinfang Zhang, Jun Jiang, Chen Tan, Haojie Li, Daozong Chen, Liangcai Jiang

**Affiliations:** ^1^ Environment-Friendly Crop Germplasm Innovation and Genetic Improvement Key Laboratory of Sichuan Province, Crop Research Institute, Sichuan Academy of Agricultural Sciences, Chengdu, China; ^2^ College of Life Sciences, Ganzhou Key Laboratory of Greenhouse Vegetable, Gannan Normal University, Ganzhou, China

**Keywords:** *Brassica napus*, anthocyanins, flower color, RNA-seq, *F3H*

## Abstract

*Brassica napus* is one of the most important oil crops in the world. Breeding oilseed rape with colorful flowers can greatly enhance the ornamental value of *B. napus* and thus improve the economic benefits of planting. As water-soluble flavonoid secondary metabolites, anthocyanins are very important for the synthesis and accumulation of pigments in the petals of plants, giving them a wide range of bright colors. Despite the documentation of over 60 distinct flower shades in *B. napus*, the intricacies underlying flower color variation remain elusive. Particularly, the mechanisms driving color development across varying flower color backgrounds necessitate further comprehensive investigation. This research undertook a comprehensive exploration through the integration of transcriptome and metabolome analyses to pinpoint pivotal genes and metabolites underpinning an array of flower colors, including beige, beige-red, yellow, orange-red, deep orange-red, white, light-purple, and purple. First, we used a two-way BLAST search to find 275 genes in the reference genome of *B. napus* Darmor v10 that were involved in making anthocyanins. The subsequent scrutiny of RNA-seq outcomes underscored notable upregulation in the structural genes *F3H* and *UGT*, alongside the *MYB75*, *GL3*, and *TTG1* transcriptional regulators within petals, showing anthocyanin accumulation. By synergizing this data with a weighted gene co-expression network analysis, we identified *CHS*, *F3H*, *MYB75*, *MYB12*, and *MYB111* as the key players driving anthocyanin synthesis in beige-red, orange-red, deep orange-red, light-purple, and purple petals. By integrating transcriptome and weighted gene co-expression network analysis findings with anthocyanin metabolism data, it is hypothesized that the upregulation of *MYB75*, which, in turn, enhances *F3H* expression, plays a pivotal role in the development of pigmented oilseed rape flowers. These findings help to understand the transcriptional regulation of anthocyanin biosynthesis in *B. napus* and provide valuable genetic resources for breeding *B. napus* varieties with novel flower colors.

## Background

*Brassica napus* (AACC, 2*n* = 38) is one of the most important oil crops in the world, which is an allotetraploid species formed by doubling after natural crosses between diploid *Brassica rapa* (AA, 2*n* = 20) and *Brassica oleracea* (CC, 2*n* = 18). After ~7,500 years of natural evolution (Chalhoub et al., 2014), *B. napus* has formed a series of variation types, such as the leaves, stems, flowers, siliques, and seeds. Prior research has demonstrated that the synthesis and accumulation of secondary metabolites such as carotenoids, flavonoids (including anthocyanins, flavones, and flavonols), and betalains play a pivotal role in the natural pigmentation of plant tissues and organs (Tanaka et al., 2008; Zhao et al., 2022). Presently, the exploration of *B. napus* attributes such as leaves (Mushtaq et al., 2016; Chen D. et al., 2020; He et al., 2021), stems (Chen et al., 2022; Fu et al., 2022; Chen et al., 2023b), flowers (Hao et al., 2022; Ye et al., 2022; Chen et al., 2023b), and seed coat color (Zhang et al., 2013; Lian et al., 2017; Xie et al., 2020; Zhai et al., 2020; Chen et al., 2023a) predominantly hinges on the intricate interplay of pro-anthocyanin and anthocyanin biosynthesis and accumulation pathways. Owing to the complex genome composition of *B. napus*, the transcriptional regulation mechanism of proanthocyanins and anthocyanins has not yet been clarified.

Anthocyanins, belonging to the secondary metabolites of flavonoids, encompass a range of compounds including cyanidin, delphinidin, pelargonidin, peonidin, malvidin, and petunidin (Tanaka et al., 2008; Zhao et al., 2022). These compounds are commonly present in plants as glycosides, imparting hues of red, purple, and blue to various plant tissues or organs (Tanaka et al., 2008; Zhao et al., 2022). Anthocyanins fulfill diverse roles, such as offering antioxidant properties, shielding against UV radiation, modulating auxin transport, serving as physiological defenses against both biotic and abiotic stresses, and acting as signals to attract pollinators and seed dispersers (Niovi Jones and Reithel, 2001; Grotewold, 2006; Tohge et al., 2017). Moreover, for human consumption, dietary intake of anthocyanins is not only a secure and non-toxic natural food coloring agent but also an effective scavenger of free radicals, thereby potentially contributing to the prevention of certain cancers, cardiovascular disorders, and other chronic ailments (Bendokas et al., 2020; Mattioli et al., 2020; Speer et al., 2020). Anthocyanin biosynthesis initiates from phenylalanine, progressing through various stages to yield compounds like delphinidin and other colored anthocyanins. This intricate biosynthetic pathway involves the orchestrated catalytic actions of numerous structural proteins. As research has evolved, the anthocyanin biosynthesis pathway has been categorized into early synthetic structural genes and late synthetic structural genes based on the sequence of enzyme modifications (Tanaka et al., 2008; Xu et al., 2013; Li et al., 2014; Xu et al., 2015). The early synthetic genes encompass *CHS*, *CHI*, *F3H*, *F3’H*, and *F3’5’H* (Shi and Xie, 2014). On the other hand, the late synthesis genes involve *DFR*, *LDOX*, and *UGT* (Shi and Xie, 2014). Regulation of the early structural genes in the anthocyanin synthesis pathway is primarily orchestrated by single or multiple MYB transcription factors. Conversely, the late synthetic structural genes are predominantly governed by the MYB-bHLH-WD40 (MBW) complex (Dubos et al., 2010; Xu et al., 2013). Contemporary investigations underscore that transcriptional regulation serves as a pivotal determinant in anthocyanin synthesis and accounts for the divergent anthocyanin accumulation observed across distinct plant tissues.

*B. napus* has undergone a series of natural variations, artificial selections, and interspecific hybridizations, leading to the generation of a diverse spectrum of germplasm resources exhibiting color variations, ranging from leaves to seeds. This repository of variation serves as invaluable material for delving into the intricate mechanisms governing anthocyanin transcriptional regulation in *B. napus*. Notably, prior investigations have pinpointed key regulatory players in anthocyanin synthesis in purple leaf *B. napus*, such as *BnaA.PL1* (Li et al., 2016) and *BnaPAP2.A7* (Chen D. et al., 2020). Furthermore, through a holistic analysis of transcriptomic and metabolomic data, researchers have identified genes like *DFR*, *ANS*, *UFGT*, and *TT19* to be pivotal components participating in the anthocyanin biosynthetic pathway within purple leaf *B. napus* (Goswami et al., 2018; He et al., 2021; Li et al., 2022). In the context of stems, a comprehensive analysis combining transcriptomic and metabolomic approaches has identified *BnaA07.PAP2* and *BnaC06.PAP2* as pivotal regulators of anthocyanin synthesis (Fu et al., 2022). Furthermore, precise mapping efforts have pinpointed *BnaPAP2.C6a* as a crucial transcription factor governed by purple stem anthocyanins (Chen et al., 2023b). Within the realm of seeds, numerous genes have been implicated in orchestrating seed coat color formation. Notably, *BnTT10* (Zhang et al., 2013), *BnTT1* (Lian et al., 2017), *BnTT2* (Xie et al., 2020), *BnTT8* (Zhai et al., 2020), *TT2*, and *MYB5* (Chen et al., 2023a) are among the reported key contributors. In the domain of petals, a diverse array of over 60 flower colors, spanning from white to dark red and mottled hues, has been sequentially documented (Xiao et al., 2022). *BnaC3.CCD4* (Zhang et al., 2015), *BnaA09.ZEP*, *BnaC09.ZEP* (Liu et al., 2020), *BnaA03.ANS* (Hao et al., 2022), *BnaA07.PAP2^In-184-317^* (Ye et al., 2022), *BnaPAP2.A7b* (Chen et al., 2023b), and *BnF3 ‘H* (Li et al., 2023) have emerged as central players governing flower color determination in *B. napus*. However, despite these advances, the intricate mechanism underpinning the rich spectrum of flower color variations in *B. napus* remains to be fully elucidated. Notably, the interplay between different genetic backgrounds and its consequential impact on flower color variation has yet to be extensively explored in the existing literature.

In this study, in order to explore the molecular mechanism of flower coloration under different flower color backgrounds, we selected *B. napus* petals with eight flower colors under white, yellow, and beige backgrounds for transcriptome and metabolome analysis. Our objective is to systematically identify genes associated with anthocyanin biosynthesis in *B. napus* and conduct comparative transcriptome and metabolome analyses as well as weighted gene co-expression network analysis (WGCNA) joint analysis. Through this approach, we aim to elucidate the key genes that govern flower color variation in colored cauliflower and investigate the general or unique mechanisms underlying the formation of flower color variation in the presence of white, yellow, and beige flower colors. Our findings indicated that the increased expression of the essential structural gene *F3H* in the anthocyanin biosynthetic pathway, along with the transcription factors *MYB75*, *GL3*, and *TTG1* of the MBW transcriptional regulatory complex, played a crucial role in the regulation of anthocyanin production and accumulation. In summary, this research will offer novel insights into the molecular mechanisms underlying flower color variation in diverse *B. napus* varieties, serving as a valuable resource for investigating the transcriptional regulation of anthocyanins across various genetic backgrounds affecting flower coloration.

## Materials and methods

### Plant materials

In this study, eight *B. napus* lines (CQY106, white flower; CQY207, light-purple flower; CQY286, purple flower; CQY112, yellow flower; CQY311, orange-red flower; CQY357, deep orange-red flower; CQY40, beige flower; CQY439, beige-red flower) with different flower colors under white, yellow, and beige backgrounds were used for transcriptome and metabolome analysis. The eight *B. napus* inbred line materials used in this study are unique to the Crop Research Institute of the Sichuan Academy of Agricultural Sciences and are not stored in a public herbarium. All lines were planted in the research field of the Sichuan Academy of Agricultural Sciences, Chengdu, China, during the 2016–2017 cropping season. The petals of half-open flowers collected on the same day at full-bloom stage with three biological replicates were immediately frozen in liquid nitrogen and then stored at -80°C for RNA and metabolite extraction.

### Metabolite extraction and profiling

Biological samples were freeze-dried with a vacuum freeze-dryer (Scientz-100F). The freeze-dried samples were crushed using a mixer mill (MM 400, Retsch) with a zirconia bead for 1.5 min at 30 Hz. Lyophilized powder (50 mg) was dissolved with 1.2 mL 70% methanol solution, vortexed six times for 30 s every 30 min, and then placed in a refrigerator at 4°C overnight. Following centrifugation at 12,000 rpm for 3 min, the extracts were filtrated (SCAA-104, 0.22-µm pore size; ANPEL, Shanghai, China, http://www.anpel.com.cn/) before UPLC-MS/MS analysis.

An ACQUITY UHPLC system (Waters Corporation, Milford, MA, USA) coupled with an AB SCIEX Triple TOF 5600 system (AB SCIEX, Framingham, MA, USA) was used to analyze the metabolic profiling in ESI positive ion modes. An ACQUITY UPLC BEH C18 column (1.7 μm, 2.1 mm × 100 mm) was employed in positive modes. The binary gradient elution system consisted of mobile phase A (0.1% formic acid in deionized water) and mobile phase B (0.1% formic acid in acetonitrile). Mobile phase B was increased linearly from 5% at 0 min to 20% at 2 min to 25% at 4 min to 60% at 9 min to 100% at 14 min and then held at 100% for 4 min. Finally, solvent B was decreased from 100% at 14 min to 5% at 18 min and held at 5% until 19.5 min. The flow rate was maintained at 0.5 mL/min with an injection volume of 10 μL. The parameters of mass spectrometry were as follows: ion source temperature, 120°C; desolvation temperature, 550°C; sampling cone, 27 eV; extraction cone, 4 eV; and the range of m/z was set as 50–1,500.

### PCA, hierarchical cluster analysis, and Pearson correlation coefficients

The raw data were converted to common data format files. Metabolomics data were acquired using the software XCMS 1.50.1 version, which produced a matrix of features with the associated retention time, accurate mass, and chromatography. Then, all ions were normalized to the total peak area of each sample in Excel 2007 (Microsoft, USA) to achieve a minimum RS. Metabolite ions were acquired in positive ion mode and exhibited less than 30% of RSD, which displayed good reproducibility of the metabolomics method. The metabolite ions which had RSD less than 30% were used for the further data processing. Then, using the molecular weight information of 108 anthocyanins that can be qualitatively and quantitatively detected by biological companies, we extracted these anthocyanin metabolites from the petals of eight flower colors. A total of 56 metabolites can be detected in the petals of eight flower colors. These 56 metabolites were used for subsequent analyses. Principal component analysis (PCA) was performed using MetaboAnalyst 5.0 online website (https://www.metaboanalyst.ca/MetaboAnalyst/faces/home.xhtml). The parameter settings are all default parameters of the default software. For details of the analysis methods, see Fu et al. (Fu et al., 2018).

### Anthocyanin biosynthetic-related gene identification in *B. napus*

Here anthocyanin biosynthesis-related genes in *B. napus* were identified by two-way BLAST and synteny analysis with *Arabidopsis thaliana*. All the protein and CDS sequences of *A. thaliana* were downloaded from TAIR (https://www.arabidopsis.org/), and the protein and CDS sequences of *B. napus* (Darmor-v10) were downloaded from BnIR (https://yanglab.hzau.edu.cn/BnIR). Then, local BLASTP and local BLASTN were used to search for the anthocyanin biosynthesis-related homologous genes of Darmor-v10 with E < 1e - 20, screen for candidate genes with consistency >65% and coverage >60%, and then remove genes that do not meet the requirements.

### RNA-seq data analysis

HISAT2 software (v2.1.0) (Pertea et al., 2016) was used to map the RNA-seq reads to the reference genome of *B. napus* (Darmor-v10, https://yanglab.hzau.edu.cn/BnIR), and then StringTie (v2.1.1) (Pertea et al., 2016) was used to obtain millions of fragments per thousand bases (FPKM) values. Excel was used to draw the expression histogram, and TBtools software (v1.133) (Chen C. et al., 2020) was used to draw the heat map. The differential expression analysis was meticulously executed employing R package DESeq2 (Love et al., 2014). Genes with a Q value ≤0.01 were deemed significant, with a further criterion of |log_2_FC| > 2. Concurrently, a comprehensive functional enrichment analysis was undertaken, encompassing Gene Ontology (GO) and Kyoto Encyclopedia of Genes and Genomes (KEGG). For the GO functional enrichment and KEGG pathway analysis, we harnessed the capabilities of Goatools (https://github.com/tanghaibao/Goatools) and KOBAS (http://kobas.cbi.pku.edu.cn/home.do), esteemed tools known for their effectiveness in uncovering key insights into gene function and pathway associations.

### Construction of co-expression network modules using association analysis modules and phenotypes

To explore the metabolite fluxes of the anthocyanin-regulated biosynthetic pathway, we used weighted gene co-expression network analysis (WGCNA) to construct a gene co-expression network for anthocyanin transcriptional regulation in different tissues of *B. napus*. Co-expression networks were constructed using the WGCNA software package in version R-4.3.1. The correlation between each co-expression module and the collected data on eight *B. napus* tissues was calculated through a correlation analysis. A co-expression network module was constructed using anthocyanin-related gene expression and 56 detectable and quantitative anthocyanin content changes. For the detailed methods, please refer to the article by Cheng et al. (Cheng et al., 2020).

## Results

### Phenotypic characterization of colored flower *B. napus*

**Figure 1:**
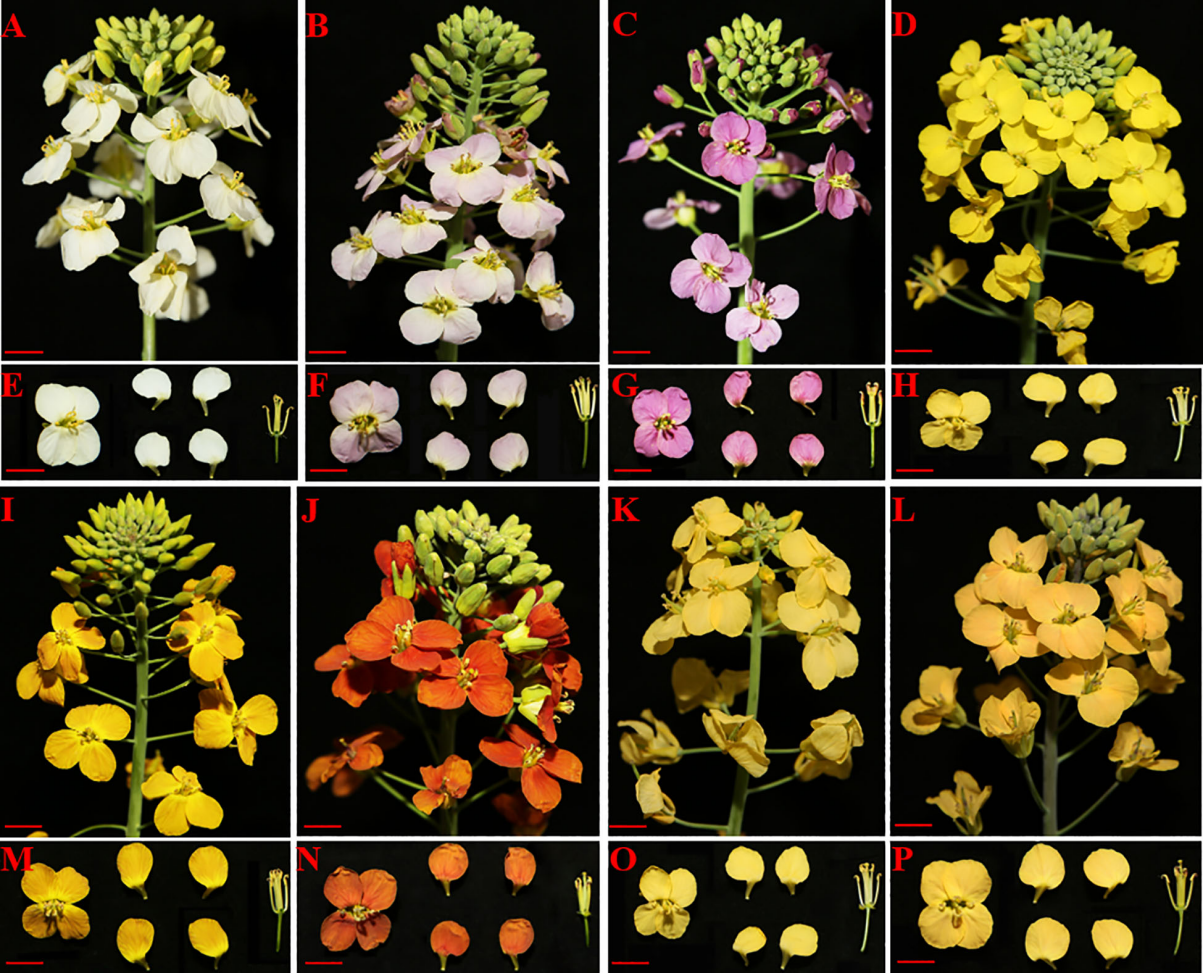
Representative phenotype of white **(A, E)**, light-purple **(B, F)**, purple **(C, G)**, yellow **(D, H)**, orange-red **(I, M)**, deep orange-red **(J, N)**, beige **(K, O)**, and beige-red **(L, P)** flower colors. Bars with red lines represent 1 cm.

During the flowering stage of *B. napus*, we characterized inflorescence phenotypes of eight different colors. The inflorescence of white appears green, with white petals after opening (**Figure 1A**) and yellow anthers (**Figure 1E**). Light-purple has light-purple petals after opening (**Figure 1B**) and red-purple anthers (**Figure 1F**). Purple flowers display purple petals after opening (**Figure 1C**) and purple anthers (**Figure 1G**). Yellow flowers are bright yellow when they open (**Figure 1D**), with both petals and anthers in yellow (**Figure 1H**). Orange-red flowers exhibit an obvious accumulation of red anthocyanins in the transporting tissue after opening (**Figure 1I**), and the anthers are orange-red (**Figure 1M**). Deep orange-red flowers appear darker after opening (**Figure 1J**), with dark orange-red petals and orange-red anthers (**Figure 1N**). Beige flowers open in apricot yellow (**Figure 1K**), with petals and anthers in yellow (**Figure 1O**), while beige-red flowers open in apricot red (**Figure 1L**), and there are noticeable anthocyanins in the petal conduction tissue, with reddish anthers (**Figure 1P**). The performance results demonstrate that as the anthocyanin content increases, this results in darker deep orange and purple petals (**Figure 1**). Additionally, we observed that anthocyanins in *B. napus* petals were specifically synthesized before the petals opened, with varying accumulations in different backgrounds (**Figure 1**). Interestingly, varying flower coloration was observed under the three distinct backgrounds, attributed to the differential accumulation of anthocyanins, showing purple on white background and on red-yellow and beige background (**Figure 1**).

### Anthocyanin metabolites in the flowers of *B. napus*

**Figure 2:**
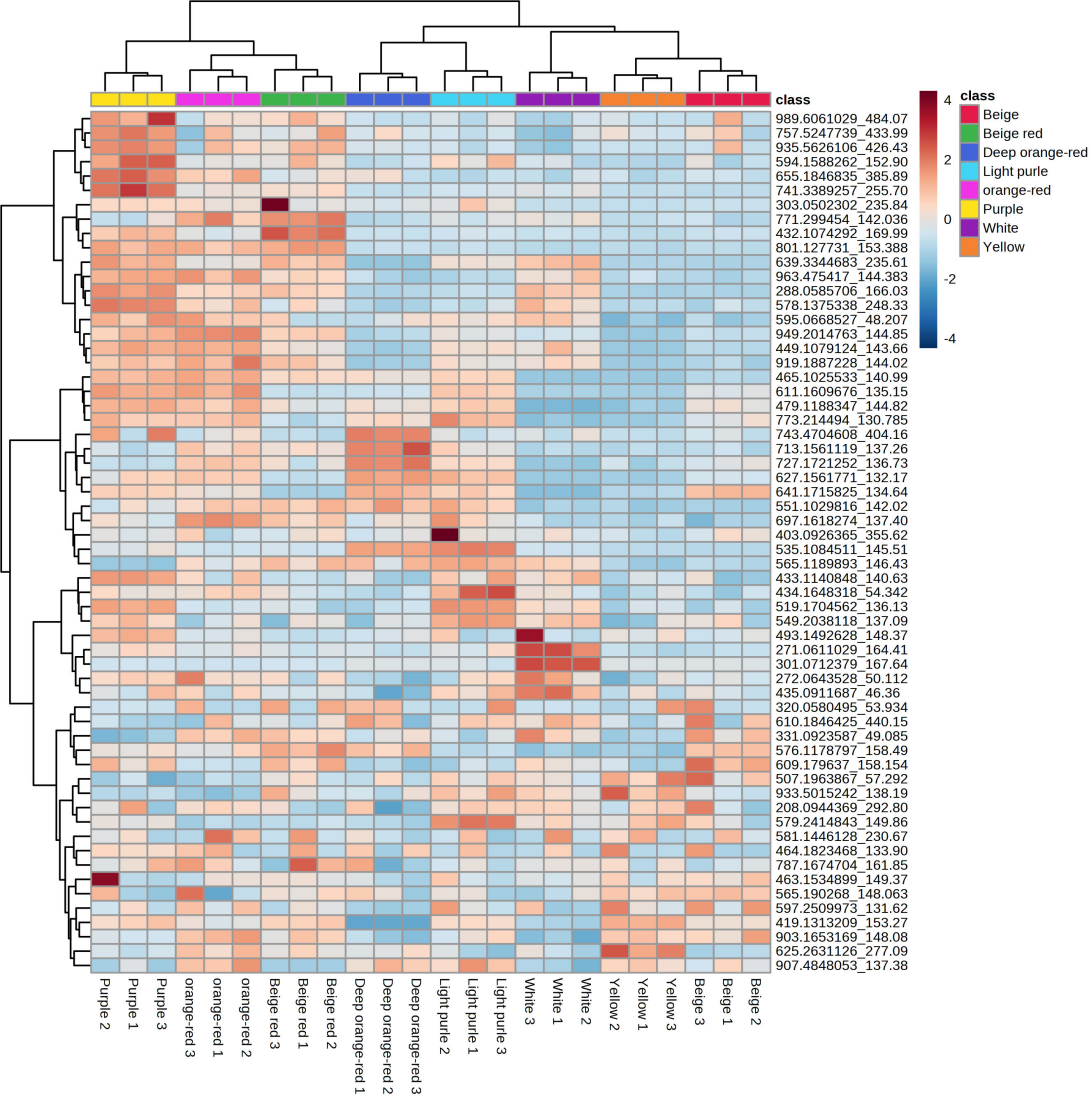
Heat map of the differential anthocyanins of eight kinds of *B. napus* flower colors.

To deeply analyze the metabolite content of eight kinds of *B. napus* flowers, we used UPLC-MS/MS to carry out an extensive targeted analysis of the metabolites contained in the petals of eight kinds of *B. napus*. The analysis revealed a total of 11,081 metabolites in the petals of the eight differently colored flowers of *B. napus* species (**Supplementary Table S1**, **Supplementary Figure S1**). Previous studies have found that the biosynthesis and accumulation of anthocyanins are the main substances responsible for the coloration of *B. napus* petals (Fu et al., 2018; Ye et al., 2022). Therefore, we extracted 56 of the 108 qualitatively and quantitatively detectable anthocyanins from eight kinds of flower metabolites (**Supplementary Table S2**). Notably, the PCA demonstrated a robust clustering of the biological replicates within each of the eight flower color groups (**Supplementary Figure S2**). Furthermore, it is interesting to observe that, among the samples with substantial anthocyanin content, those with purple and orange coloration exhibited similar clustering patterns, while the same was observed for samples displaying light-purple, beige, and deep orange-red hues (**Supplementary Figure S2**). Upon subjecting the petal metabolites of the eight distinctly colored *B. napus* flower varieties to dendritic clustering analysis, a compelling pattern emerged. Notably, this analysis revealed that the three biological replicates of each flower color exhibited a tendency to cluster together, underscoring the reproducibility of our findings. Within this context, it is noteworthy that under the white background condition, white flowers, along with light purple and purple variants, shared a branch. Intriguingly, within the subset of flower color samples, those displaying beige, yellow, white, and deep orange red hues formed a distinct branch. Similarly, orange-red, beige-red, purple, and light purple flower varieties demonstrated co-clustering on another branch (**Supplementary Figure S3**). Furthermore, delving into the metabolite content analysis through a heat map approach, we observed a partitioning of the petal metabolites of the eight flower colors into three prominent clusters. Additionally, the clustering pattern of the metabolites mirrored the branching structure of a developmental tree. However, it is important to highlight that even within the same branch, the content of metabolites exhibited significant variability (**Figure 2**). Notably, a distinct trend emerged in terms of metabolite abundance among the various flower color categories. Specifically, petals exhibiting shades of purple, beige-red, and orange-red showcased comparable levels of a larger set of metabolites. Conversely, a similar observation was made for petals displaying shades of deep orange red, light purple, white, yellow, and beige, where these variants also shared similar quantities of a distinct subset of metabolites (**Figure 2**).

### Comparative transcriptome analysis of *B. napus* with different flower colors

**Figure 3:**
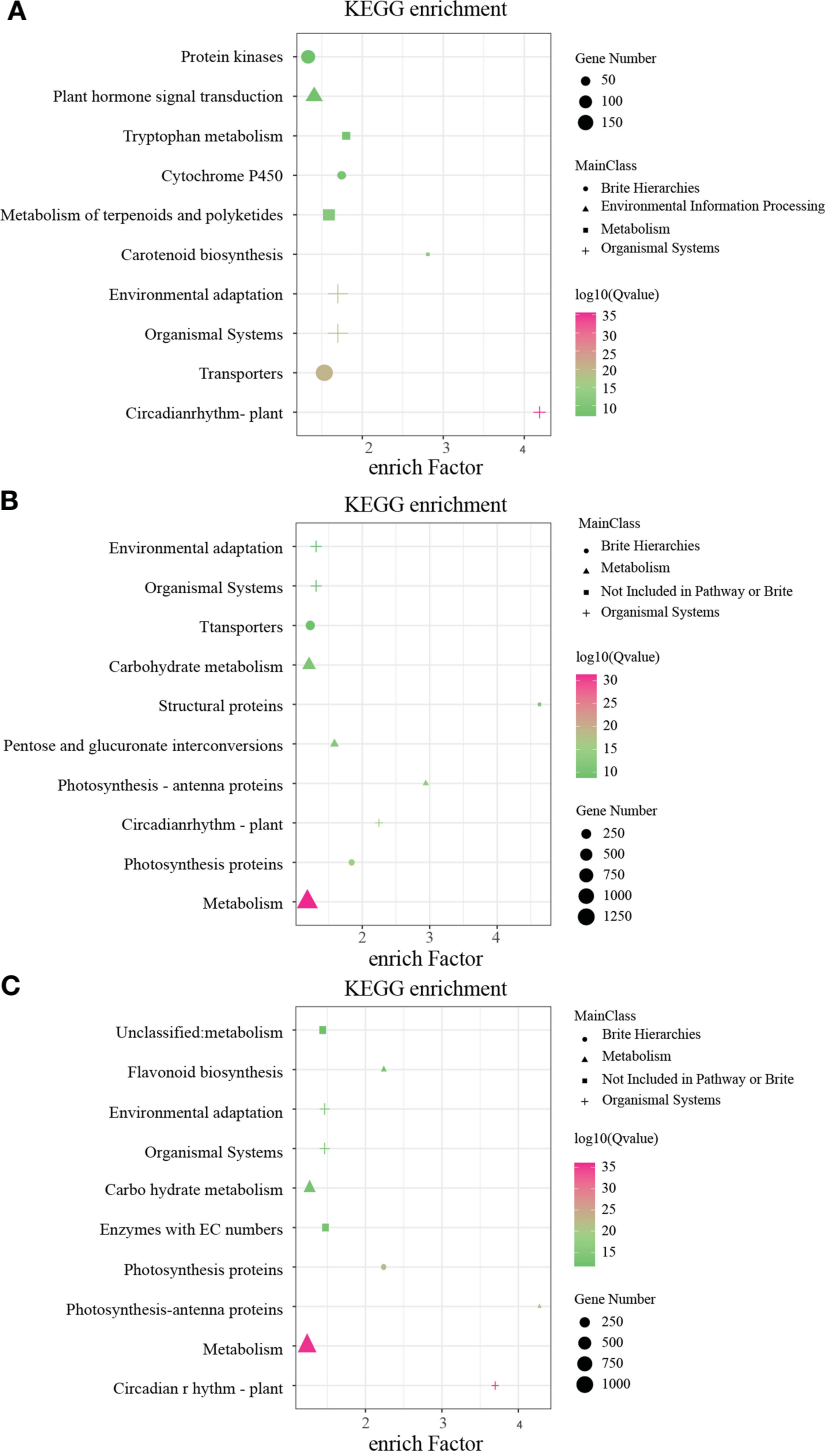
Kyoto Encyclopedia of Genes and Genomes (KEGG) pathway enrichment annotation of differentially expressed genes (DEGs) in white, yellow, and beige genetic background. **(A)** KEGG pathway enrichment of DEGs between beige and beige-red in beige background. **(B)** KEGG pathway enrichment of DEGs between yellow, orange-red, and deep orange-red in yellow background. **(C)** KEGG pathway enrichment of DEGs between white, light-purple, and purple in yellow background.

In order to identify key genes regulating anthocyanin synthesis and elucidate the molecular basis of flower color formation in *B. napus* under white, yellow, and beige backgrounds, RNA-seq analysis was performed on unopened petals of eight flower colors. The results of differentially expressed gene (DEG) analysis showed that there were 5,239 DEGs in beige vs. beige-red, 6,587 DEGs in yellow vs. orange-red, 5,123 DEGs in yellow vs. deep orange-red, 3,437 DEGs in orange vs. deep orange, 2,577 DEGs in white vs. light-purple, 6,253 DEGs in white vs. purple has, and 6,253 DEGs in light-purple vs. purple (**Supplementary T** **able S3**). Subsequently, we performed KEGG annotation analysis on these differentially expressed genes, and the KEGG annotation results of DEGs under the three flower color backgrounds were highly enriched in the Brite Hierarchies, Metabolism, Not Included in Pathway or Brite, Organismal Systems, and Environmental Information Processing pathways (**Figure 3**). The DEGs of the three flower color backgrounds have a strong response in the Circadian Rhythm–Plant pathway (**Figure 3**), while the number of genes enriched in the Metabolism pathway is the largest under the yellow and white backgrounds (**Figures 3A, B**), and the beige and beige differential genes have a strong enrichment response in the Carotenoid Biosynthesis pathway (**Figure 3C**).

### Identification of anthocyanin biosynthesis-related genes in *B. napus*

**Figure 4:**
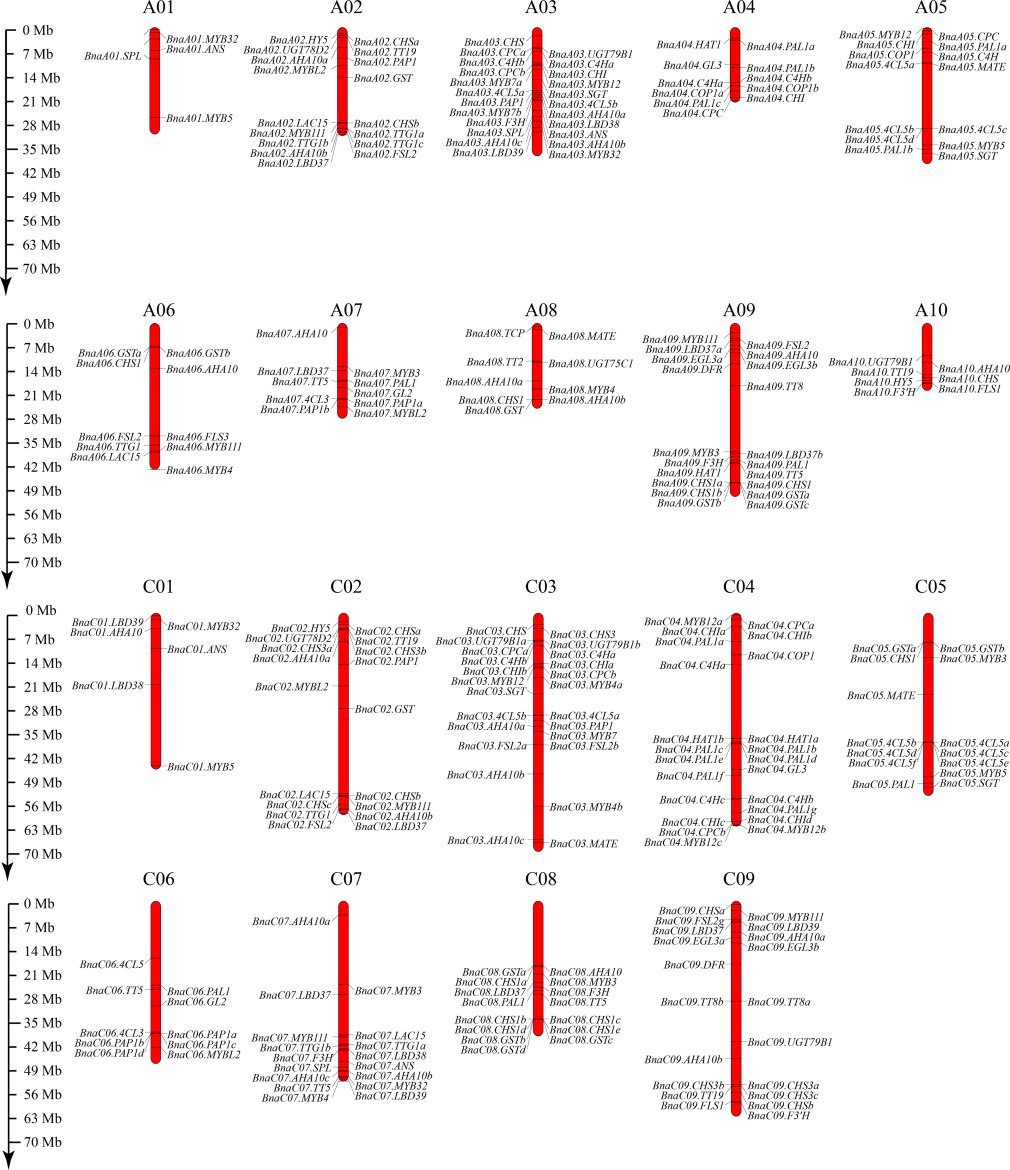
Distribution of 275 anthocyanin-related genes on the 19 chromosomes of *B. napus*. The bars indicate the chromosomes of *B. napus*, and the relative positions of anthocyanin-related genes were marked on the chromosomes. The scale ruler on the left side shows the physical distance of the chromosomes.

To better analyze the expression patterns of anthocyanin-related genes in the eight flower colors, we used 52 genes related to the anthocyanin synthesis pathway in *A. thaliana thaliana* as seeds, and 275 genes related to anthocyanin synthesis were identified from *B. napus* Darmor v10 reference genome (**Supplementary Table S4**). The anthocyanin-related genes were distributed on 19 pairs of chromosomes in *B. napus*, the least being six (A01 and C01 chromosomes) and the most being 24 (C03 chromosome) (**Figure 4**). The gene with the largest number of copies was *PAL1*, reaching 17, while no homologous gene was identified in *MYB113* and *MYB114*. Interestingly, 10 homologous copies of *PAP1*(*MYB75*) and *PAP2*(*MYB90*) were identified. Due to the high homology of the two genes, they are collectively referred to as *PAP1*(*MYB75*). Interestingly, *PAP1*(*MYB75*) has two tandem duplications on chromosome A7 and four tandem duplications on chromosome C6 (**Figure 4**).

### Screening of differentially expressed genes related to anthocyanin biosynthesis

To delve further into the intricate molecular mechanisms governing the synthesis and accrual of pivotal metabolites like anthocyanins under the purview of the three distinct flower color backgrounds, we harnessed the *B. napus* reference genome, with 52 anthocyanin-related genes sourced from *A. thaliana* serving as the seed sequences. From this composite approach, we successfully pinpointed 274 genes linked to anthocyanin biosynthesis within the *B. napus* genome, utilizing *B. napus* Darmor-10 as the central reference (**Supplementary Table S5**). Subsequently, our focus pivoted to the exploration of anthocyanin-associated DEGs, leveraging the RNA-seq data. This meticulous analysis brought to light a spectrum of differentially expressed genes, wherein 39 DEGs were identified in the beige vs. beige-red comparison, followed by 45 DEGs in the yellow vs. orange-red scenario, 17 DEGs in the yellow vs. deep orange-red contrast, and 18 DEGs in the red vs. deep orange comparison. Further comparisons led to the identification of 11 DEGs in the white vs. light-purple scenario, 59 DEGs in the white vs. purple context, and, finally, 51 DEGs within the light-purple vs. purple comparison (**Supplementary T** **able S6**). In this context, an array of pivotal players has emerged. Structural genes, including *CHS*, *CHI*, *DFR*, *ANS*, and *UGT*, along with transcription factors like *PAP1* (*MYB75*) and glutathione S-transferase *TT19*, have been discerned as exhibiting differential expression across three distinct genetic backgrounds. This observation underlines the centrality of anthocyanin synthesis and accumulation in governing the shift from red to purple hues in floral pigmentation.

### Expression analysis of genes in the anthocyanin biosynthetic pathway in the flowers of *B. napus*

**Figure 5:**
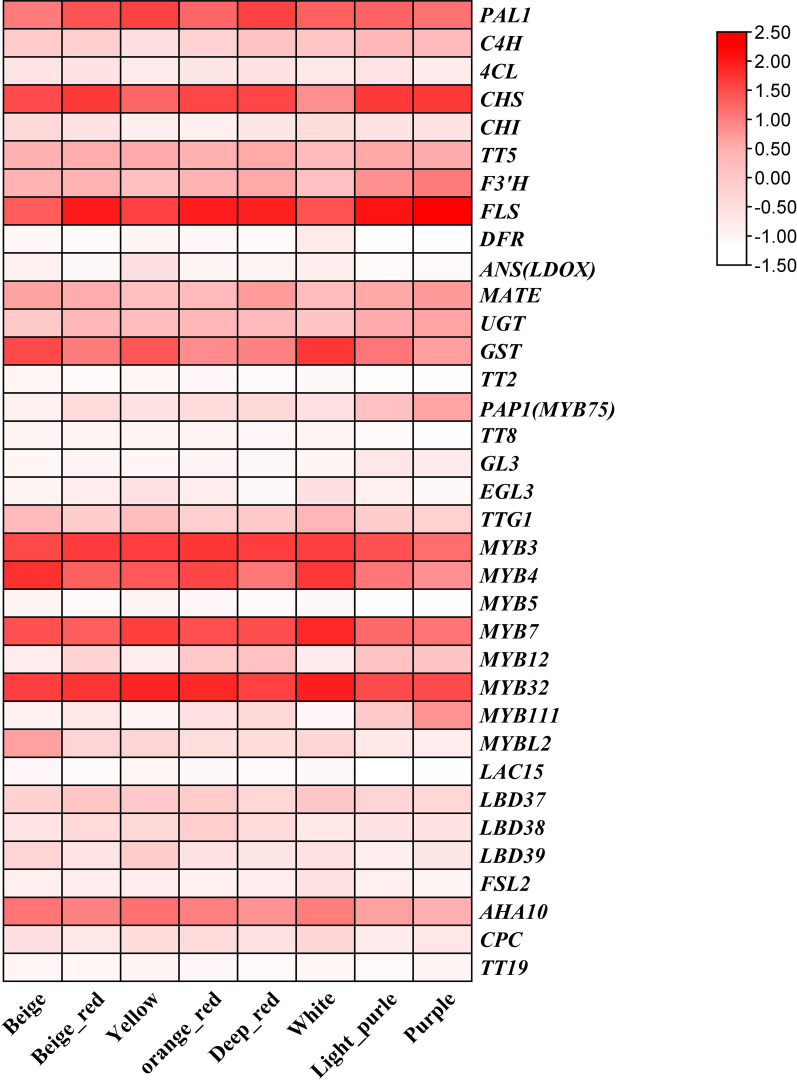
The expression patterns of anthocyanin-related genes in eight colors of *B. napus* were analyzed using a heat map that represents the fragments per thousand bases values of these genes. The colors on the heat map, ranging from red to pink and white, indicate the expression levels from high to low.

In order to further explore the expression patterns of anthocyanin biosynthesis-related genes under the three flower color backgrounds, we constructed a heat map of anthocyanin-related gene expression using eight sets of petal RNA-seq data (**Figure 5**). The results showed that the expression levels of structural genes *PAL*, *C4H*, *CHS*, *F3’H*, *UGT*, and *GST* and transcriptional regulatory genes *PAP1(MYB75)*, *MYB3*, *MYB4*, *MYB7*, *MYB32*, and *AHA10* related to anthocyanin biosynthesis pathway were relatively high. Previous studies have shown that *MYB3*, *MYB4*, *MYB7*, and *MYB32* mainly target and regulate the expression of *PAL*, *C4H*, and *4CL* genes in the phenylpropane synthesis pathway, while these structural genes and transcriptional regulators are highly expressed in our materials. Meanwhile, *MYB11*, *MYB12*, and *MYB111* target the structural genes *CHS*, *CHI*, and *F3’H* of the flavonoid synthesis pathway in eight petals of *B. napus*; the expression levels of these genes in beige-red, orange-red, deep orange-red, light-purple and purple petals were significantly higher than those in yellow, white, and beige. Particularly, *PAP1(MYB75)* was significantly upregulated in beige-red, orange-red, deep orange-red, light-purple, and purple petals with anthocyanin accumulation, which further suggests that *PAP1(MYB75)* is responsible for the regulation of anthocyanin biosynthesis in the petals of *B. napus*.

### WGCNA analysis of genes related to anthocyanin biosynthesis in eight different flower colors of *B. napus*

**Figure 6:**
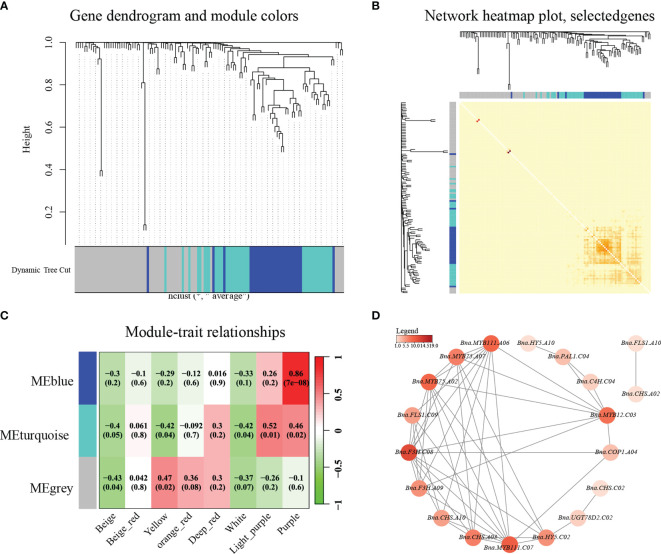
A co-expression analysis of genes related to anthocyanin biosynthesis was conducted in the eight colors of *B. napus* tissues. **(A)** Modular hierarchical clustering: The co-expression modules are depicted in different colors, while gray modules indicate no correlation between genes. **(B)** Module gene clustering heat map: The gene expression network of anthocyanin biosynthesis-related genes in different tissues was analyzed using weighted gene co-expression network analysis, leading to the clustering of genes into distinct co-expression modules. **(C)** Module-to-sample correlation heat map: A correlation analysis was performed between the co-expression modules of various genes associated with anthocyanin biosynthesis in different tissues. The numbers above the heat map indicate the Pearson correlation coefficient (*r*) values. **(D)** Cytoscape representation of the co-expression network of the hub gene with edge weights ≥0.4 in the MEblue module of a purple flower.

To discern the intricate expression relationships among genes implicated in anthocyanin biosynthesis across the diverse spectrum of eight *B. napus* flower colors, a comprehensive weighted gene co-expression network analysis (WGCNA) was undertaken. This approach facilitated the grouping of all anthocyanin-related genes into two distinct WGCNA modules, with an additional unclustered gray module observed (**Figure 6A**). The interrelation analysis within these modules demonstrated robust correlations, particularly evident in the blue modules (**Figure 6B**). Subsequently, a meticulous dissection of the intermodule correlations across the eight color variants was conducted. Intriguingly, within the MEgrey module, the beige sample displayed the highest correlation (module–sample correlation = 0.96, *p*-value = 4e-13) (**Figure 6C**). In the MEturquoise module, the correlations pertaining to petals with anthocyanin accumulation outstripped those of their non-anthocyanic counterparts, such as white, yellow, and beige (**Figure 6C**). Notably, the purple *B. napus* module exhibited the most substantial correlation (module–sample correlation = 0.74, *p*-value = 4e-05), followed by light-purple (module–sample correlation = 0.39, *p*-value = 0.06) and deep orange-red (module–sample correlation = 0.11, *p*-value = 0.6). Evidently, the outcomes of the WGCNA bolster the assertion that modules characterized by anthocyanin accumulation showcase augmented correlations while concurrently unveiling nuanced differentiations among flower colors under distinct genetic backgrounds.

In order to further determine the relationship between the genes in the module and the screening hub genes (highly connected genes), we used the blue module to construct an anthocyanin-related differentially expressed protein interaction network (**Figure 6D**). In the blue module, 19 genes related to anthocyanin biosynthesis showed a high expression of correlation, including structural proteins, transcriptional regulatory proteins, and transporters. In the interactive network diagram, the early catalytic proteins PAL1, C4H, CHS, and F3H, the transcriptional regulatory factors *MYB12*, *MYB75*, and *MYB111* in the anthocyanin synthesis pathway, the late catalytic protein UGT78D2, and the transcriptional regulatory factor *HY5* in the anthocyanin synthesis pathway have a strong regulation relationship (**Figure 6D**). These highly connected and interacting genes are called central genes in the co-expression network and play an important role in understanding the biological mechanism of anthocyanin synthesis regulation. In addition, we found that *HY5* and *COP1*, which regulate the synthesis of anthocyanins by the plant light signal pathway, have strong interactions with *PAL1*, *CHS*, *F3H*, and *UGT78D2* and *MYB12*, *MYB75*, and *MYB111* in the regulation network, indicating that the light signal pathway may be involved in the colorful *B. napus* biosynthesis of anthocyanins. Importantly, our results indicated a strong interaction between *F3H* and *MYB12*, *MYB75*, *MYB111*, and *HY5* in *B. napus* (**Figure 6D**).

## Discussion

*B. napus* is a widely cultivated oilseed crop in the world. In China, in order to further improve the economic benefits of *B. napus* planting, the ornamental value of *B. napus* is increasingly of concern by the majority of breeders (Xiao et al., 2022). In this study, we jointly analyzed the metabolites and anthocyanin-related gene expression patterns of eight flower colors in *B. napus* under three flower color backgrounds by using transcriptome and metabolome. The comparative analysis of transcriptomes revealed that genes exhibiting a differential expression in white and yellow backgrounds were predominantly enriched in the anthocyanin metabolic pathway, whereas the beige background displayed a pronounced enrichment in the carotenoid biosynthesis pathway. Subsequently, a weighted co-expression network of genes related to anthocyanin was constructed across the eight flower colors, with a particularly robust expression pattern observed in petals. The results of the hub gene interaction network analysis suggest that the high interconnection among *MYB12*, *MYB75*, *MYB111*, *CHS*, and *F3H* plays a crucial role in regulating the biosynthesis of anthocyanins in colored *B. napus*. Additionally, our findings indicate that the light signaling pathway proteins *HY5* and *COP1* exhibit strong interactions with the MBW transcription complex and *F3H*, suggesting a potential regulatory role of light signals in anthocyanin biosynthesis.

In recent years, researchers have used biological methods such as multi-omics joint analysis, fine mapping, and functional verification to study the coloration mechanism of yellow and white flowers, the transcriptional regulation mechanism of apricot and pink flowers, the variation mechanism of orange-red flower color and the coloration of purple *B. napus*; the mechanism has been extensively studied. Zhang et al. (Zhang et al., 2015) fine-mapped the key gene *BnaC3.CCD4* that regulates *B. napus* yellow/white flowers. A functional analysis found that the functional silencing of different types of *CCD4* mutations is the key to the formation of yellow flowers. However, *BnaA09.ZEP* and *BnaC09.ZEP* regulate the biosynthesis and accumulation of carotenoids, which is the key to the coloration of orange-yellow *B. napus* flowers (Liu et al., 2020). Hao et al. (Hao et al., 2022) found that *BnaA03.ANS* is a key gene regulating red-flowered *B. napus* by the combined analysis of transcriptome and metabolism. RNA interference of *BnaA03.ANS* in red *B. napus* altered the petal colors from raspberry red to beige red and zinc yellow under different interference levels. Ye et al. (Ye et al., 2022) fine-mapped the key gene *BnaA07.PAP2* that regulates apricot and pink; a functional verification found that the insertion of 210 and 412 bp in the promoter region is the key to activate the expression of *BnaA07.PAP2*. Chen et al. (Chen et al., 2023b) used orange red-flowered and white-flowered *B. napus* No 2127 to construct a mapping population and fine-mapped the key gene *BnaPAP2.A07b* that regulates orange safflower. Li et al. (Li et al., 2023) used the combined analysis of transcriptome and metabolome to find that *BnF3’H* is a key gene regulating the purple color of *B. napus* petals. Interestingly, our research is the first to report that *F3H* is a key gene that regulates the formation of colorful flowers in *B. napus*, which, together with the previously reported *BnaA03.ANS* and *BnF3’H*, further enriches the transcriptional regulation mechanism of *B. napus*. In addition, our results further verified the important role of MBW complex in the regulation of anthocyanin biosynthesis in *B. napus* petals.

The parents of *B. napus*, *B. rapa*, and *B. oleracea*, both experienced genome-wide triploidization events before natural hybridization (Cheng et al., 2013), and *B. rapa* and *B. oleracea* originated from the same parent. The genomes of these two parent species, *B. rapa* and *B. oleracea*, exhibit a close genetic affinity (Cheng et al., 2016). Following natural hybridization, *B. napus* underwent extensive chromosomal reorganization upon its doubling event, thereby introducing varying copies of homologous genes within its genome, ranging from absent to multiple instances, and even engendering the emergence of novel genes (Chalhoub et al., 2014). The multiple copies of these homologous genes have functional redundancy, resulting in functional variations such as neofunctionalization, subfunctionalization, and silencing, and some genes have also formed common and specific regulatory mechanisms. In orange-colored *B. napus* flowers, *BnaA09.ZEP* and *BnaC09.ZEP* co-regulated carotenoid biosynthesis (Liu et al., 2020), while in purple *B. napus*, transcript factors *BnaA07.PAP2* and *BnaC06.PAP2* were identified as the key to the upregulation of most of anthocyanin synthesis genes that promoted anthocyanin accumulation (Fu et al., 2022). In another purple-leaf *B. napus*, the homologous genes *BnaPAP2.A07* and *BnaPAP2.C6a* of *AtPAP2* specifically regulate the biosynthesis of anthocyanins in the leaves and stems, respectively (Chen D. et al., 2020; Chen et al., 2023b). At the same time, the mutation caused by the two insertions of *AtPAP2*’s homologous gene *BnaA07.PAP2^In-184-317^* in the promoter is considered to be the key to regulating the coloration of apricot and pink flowers (Ye et al., 2022). These findings suggest that distinct copies of *AtPAP2* within *B. napus* execute co-regulatory and tissue-specific control across diverse materials, with its precise molecular mechanism warranting a deeper investigation. Furthermore, up to 63 unique flower hues in *B. napus* have been documented to date (Xiao et al., 2022). Additionally, 63 kinds of flower colors of *B. napus* have been reported so far (Xiao et al., 2022), and the molecular mechanism of flower color variation has not yet been clarified, especially the co-coloring of anthocyanins with flavonols and carotenoids under different flower color backgrounds, and its regulatory mechanism needs to be further studied as a follow-up. In the intricate genetic context of *B. napus*, the functional divergence, transcriptional and post-transcriptional control of repetitive genes, post-translational modification of proteins, co-pigmentation of anthocyanins with flavonols, carotenoids, and metal ions, and pH alteration collectively contribute to the modulation of flower color. This phenomenon likely underlies the extensive diversity of flower color in *B. napus*, necessitating further investigation into the associated mechanisms.

## Data availability statement

The datasets presented in this study can be found in online repositories. The names of the repository/repositories and accession number(s) can be found in the article/**Supplementary Material**.

## Author contributions

CC: Writing – review & editing, Writing – original draft, Visualization, Validation, Supervision, Software, Resources, Project administration, Methodology, Investigation, Funding acquisition, Formal analysis, Data curation, Conceptualization. KZ: Writing – review & editing, Formal analysis. LC: Writing – original draft, Software, Investigation, Resources. BZ: Writing – original draft, Formal analysis, Data curation. JZ: Writing – original draft, Software. JJ: Writing – original draft, Investigation, Data curation. CT: Writing – original draft. HL: Writing – review & editing, Validation, Resources, Formal analysis. DC: Writing – review & editing, Writing – original draft, Visualization, Supervision, Software, Project administration, Methodology, Investigation, Funding acquisition, Data curation, Conceptualization, Validation, Resources, Formal analysis. LJ: Writing – review & editing, Resources, Funding acquisition.

## Glossary

**Table:**

PAL	phenylalanine ammonia-lyase
C4H	cinnamate 4-hydroxylase
4CL	4-coumarate-CoA-ligase 4
CHS	chalcone synthase
CHI	chalcone isomerase
F3H	flavanone-3-hydroxylase
F3′H	flavonoid-3′-hydroxylase
F3′5′H	flavonoid-3′,5′-hydroxylase
FLS	flavonol synthase
DFR	dihydroflavonol 4-reductase
ANS	anthocyanidin synthase
BAN	anthocyanidin oxidoreductase
AT	acyltransferase
GT	glucosyltransferase
UFGT	UDP-glucose/flavonoid glucosyltransferase
MT	methyl transferase
TTG1	transparent testa glabra 1
TT8	transparent testa 8
GL3	glabra 3
EGL3	enhancer of glabra 3
PAP1/2	production of anthocyanin pigment 1/2
MYB75/90/113/114	MYB domain protein 75/90/113/114
