# Supplementary figures and images for: Unraveling the mechanism of flower color variation in Brassica napus by integrated metabolome and transcriptome analyses
Source: Front Plant Sci. 2024 Jun 12;15:1419508. doi: 10.3389/fpls.2024.1419508 (PMC11199733; doi:10.3389/fpls.2024.1419508)

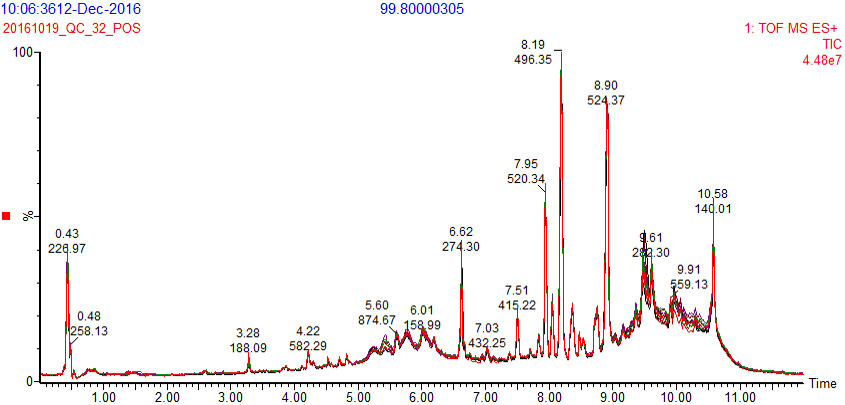

Supplement: Supplementary file 2 [file Image_1.jpeg]

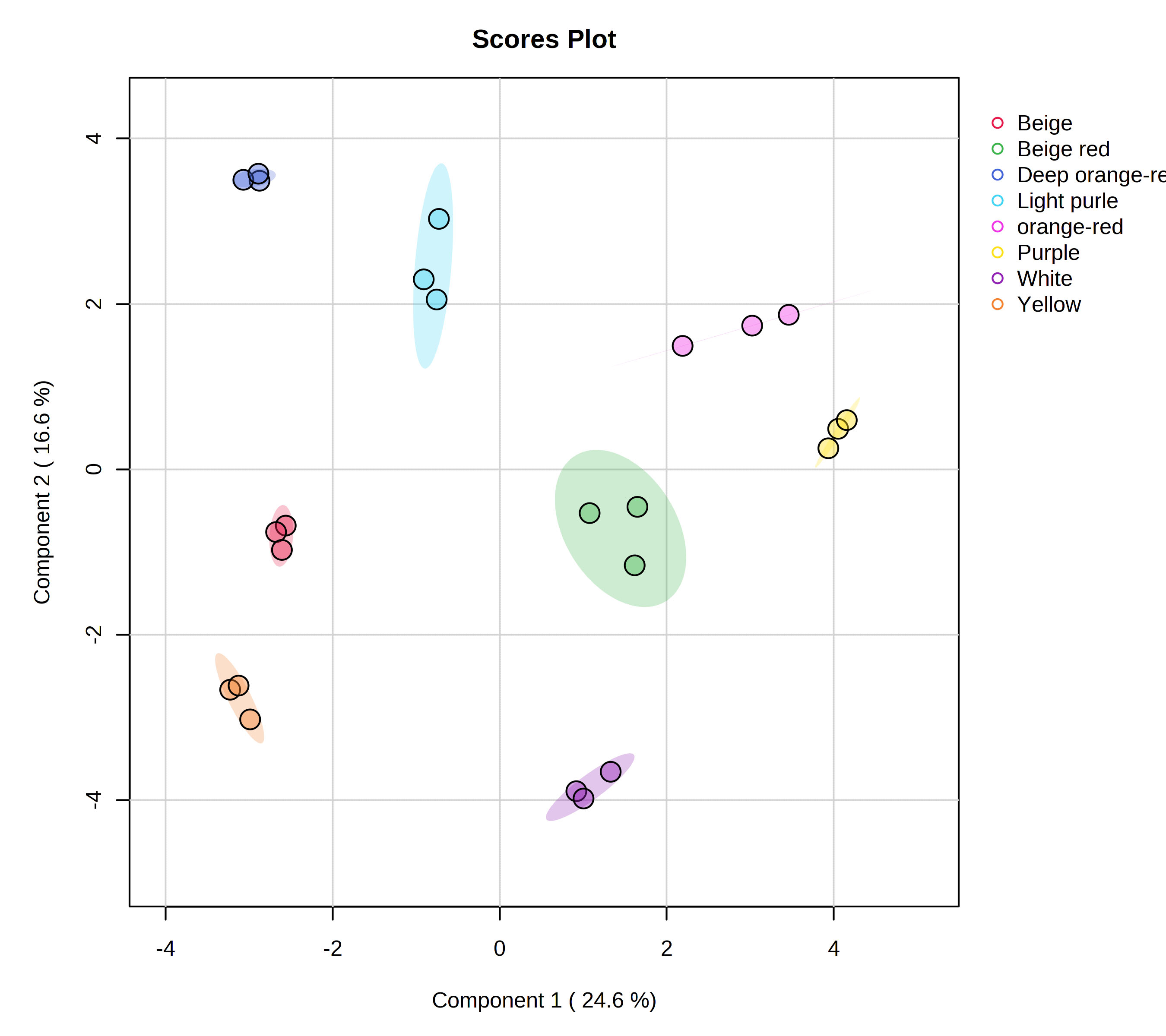

Supplement: Supplementary file 3 [file Image_2.jpeg]

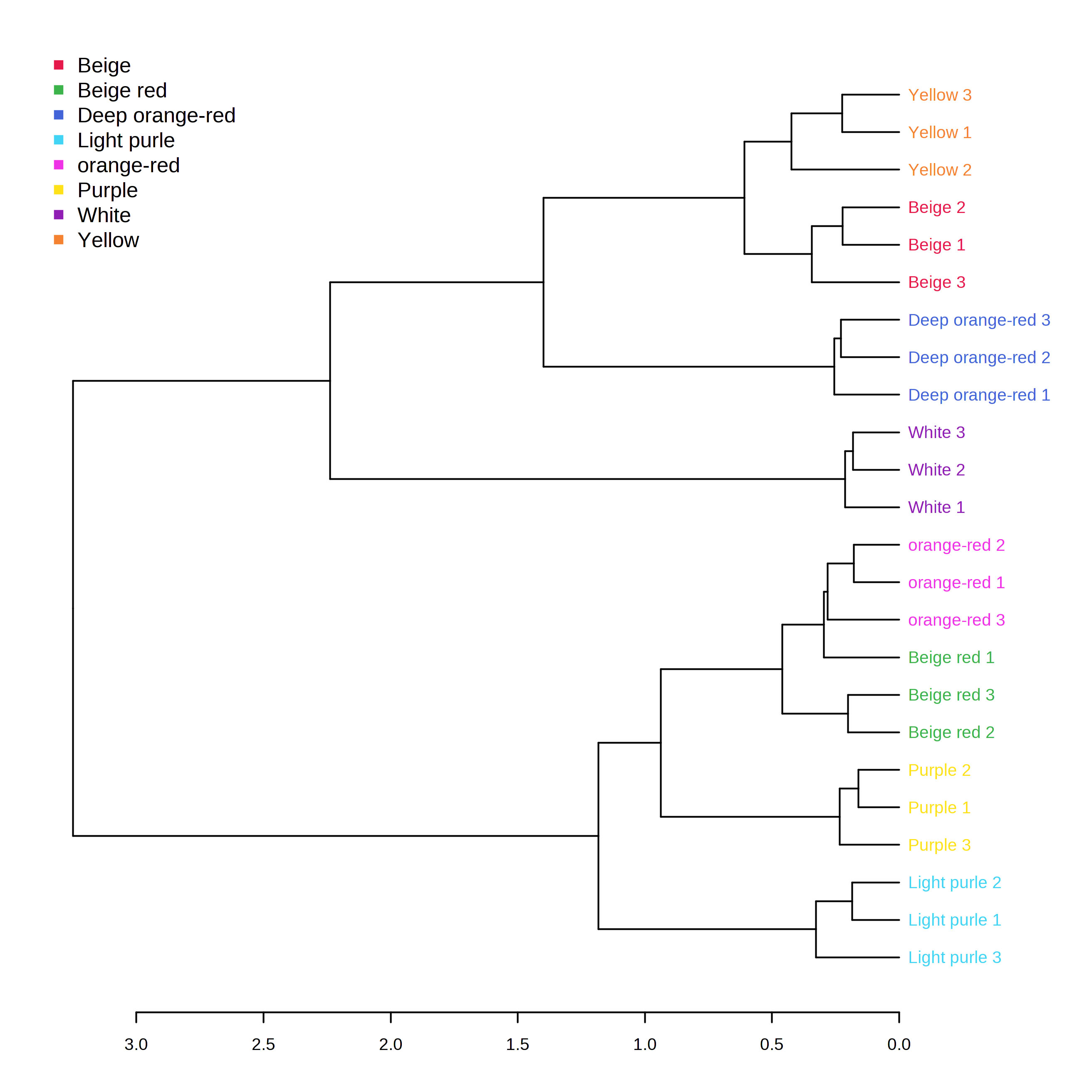

Supplement: Supplementary file 4 [file Image_3.jpeg]
